# Hepatocellular carcinoma with extrahepatic metastasis: Are there still candidates for transarterial chemoembolization as an initial treatment?

**DOI:** 10.1371/journal.pone.0213547

**Published:** 2019-03-07

**Authors:** Jihye Kim, Dong-Hyun Sinn, Moon Seok Choi, Wonseok Kang, Geum-Youn Gwak, Yong-Han Paik, Joon Hyeok Lee, Kwang Cheol Koh, Seung Woon Paik

**Affiliations:** Department of Medicine, Samsung Medical Center, Sungkyunkwan University School of Medicine, Seoul, Korea; Buenos Aires Physics Institute, ARGENTINA

## Abstract

**Background and aim:**

Currently, sorafenib is indicated for hepatocellular carcinoma (HCC) with extrahepatic metastasis (EHM), and many other systemic agents are becoming available. However, a few HCC patients with EHM still undergo transarterial chemoembolization (TACE) for intrahepatic tumor control. We aimed to investigate whether TACE is appropriate for patients with EHM, and if so, which subgroup may benefit from TACE.

**Methods:**

A total of 186 consecutive HCC patients (median: 55 years, male: 86.0%, hepatitis B virus: 81.7%, Child-Pugh Class A: 83.3%) with EHM (nodal metastasis: 60.8%, distant metastasis: 39.2%) between 2010 and 2014 were analyzed. Initial treatment included sorafenib in 69 patients, and TACE in 117 patients.

**Results:**

During a median follow-up of 6.6 months (range: 0.2–94.6 months), mortality was observed in 90.3% (168/186). The median survival was better for patients who received TACE than those treated with sorafenib (8.2 months vs. 4.6 months, p < 0.001). However, baseline characteristics varied between patients initially treated with TACE and sorafenib, and the treatment modality was not an independent factor associated with overall survival (hazard ratio: 1.19, 95% confidence interval: 0.81–1.75, p = 0.36). In sub-group analysis, TACE was associated with better survival only among younger patients and those with segmental/lobar portal vein invasion.

**Conclusion:**

In HCC patients with EHM, TACE was not an independent favorable prognostic factor compared to sorafenib. The concept of intrahepatic control in HCC patients with EHM may need to be reevaluated in the era of promising systemic therapies, although there can be specific subgroups who still benefit from TACE.

## Introduction

Primary liver cancer is the sixth most commonly diagnosed cancer, with 80% of all cases manifesting hepatocellular carcinoma (HCC), and is the second most common cause of cancer death in the world [1,2]. HCC may develop extrahepatic metastasis (EHM). Lung is the most common site of metastasis in HCC, followed by lymph nodes, bones and the adrenal glands [3]. Currently, systemic agents such as sorafenib are available for the treatment of HCC patients with EHM [4,5], and can prolong survival of HCC patients [6]. However, in a subset analysis of the phase III sorafenib Asia-Pacific trial, sorafenib was associated with only modest improvement of median overall survival compared with placebo in patients with lung metastasis (5.6 versus 4.2 months), and the difference was not statistically significant [hazard ratio (HR): 0.87, 95% confidence interval (CI): 0.56, 1.37]. Similar results were found in patients with lymph node metastasis (overall survival, 5.6 versus 3.2 months, HR: 0.64, 95% CI: 0.38, 1.08) [7]. Moreover, only a minor proportion of HCC patients die from EHM, while most patients die of intrahepatic HCC progression or liver failure [8–10]. The controllability of intrahepatic lesions was identified as an important prognostic factor for survival in HCC patients with EHM [11]. Hence, in real-life practice, some patients with HCC and EHM have been treated for intrahepatic HCC using locoregional treatments, usually transarterial chemoembolization (TACE) [12–15], without robust evidence to support. Moreover, these data were derived from an era when sorafenib and other immunotherapic agents were not widely available.

The purpose of this study was to determine whether TACE was associated with improved survival compared with sorafenib in HCC patients with EHM and to identify possible subgroups of HCC patients with EHM who may benefit from TACE.

## Materials and methods

### Study design and patient selection

This single-center, retrospective cohort study was conducted using a prospectively collected HCC registry at the Samsung Medical Center, Seoul, Korea between 2010 and 2014. The HCC registry of Samsung Medical Center is a prospective registry that records baseline clinical characteristics, tumor variables, and the initial treatment modalities of every newly-diagnosed HCC patient aged 18 years or older who received care at the Samsung Medical Center. A diagnosis of HCC was established either histologically or clinically according to the regional HCC guideline [16,17]. We screened a total of 261 HCC patients with EHM at diagnosis. Among them, we included a total of 186 consecutive newly diagnosed HCC patients with EHM who received TACE or sorafenib as an initial treatment, by excluding patients who received supportive care only (n = 44) or who underwent resection, radiofrequency ablation or radiation therapy as an initial treatment (n = 31). The study was approved by the Ethics Committee of Samsung Medical Center and was conducted in accordance with the principles of the Declaration of Helsinki. Because this study was based on a retrospective analysis of existing administrative and clinical data, the requirement for informed patient consent was waived by the Institutional Review Board.

### Treatment and follow-up

In principle, sorafenib was started at a dose of 400 mg twice daily (800 mg/day). The patients were usually treated with the initial dose during the first 2 weeks, continued every 4 weeks if there were no side effects. Dose reduction or temporary interruption of sorafenib followed any significant drug-related adverse events. If the patient tolerated sorafenib, the therapy was continued until disease progression [6].

Conventional TACE was performed with an intra-arterial injection of a mixture of doxorubicin hydrochloride (Adriamycin; Dong-A Pharm, Seoul, Republic of Korea) and iodized oil (Lipiodol; Laboratoire Andre Guerbet, Aulnay-sous-Bois, France) following a femoral approach, celiac angiogram and superselection of tumor feeder at the level of the segmental or subsegmental artery with a micro-guidewire and a 2.0-Fr microcatheter. The feeder(s) were embolized with gelatin sponge pledgets (Cutanplast, MasciaBrunelli S.P.A, Milano, Italy) until hemostasis was achieved [18]. Contrast–enhanced computed tomography (CT) or magnetic resonance (MR) imaging was performed at baseline and every 2–4 months.

### Variables and data collection

We categorized patients according to initial treatment modality: sorafenib or TACE. We used the following variables obtained from the HCC registry by trained abstractors: age at diagnosis, sex, etiology of HCC, Eastern Cooperative Oncology Group (ECOG) performance status, Child-Pugh Class, serum albumin and bilirubin levels, albumin-bilirubin (ALBI) grade, serum alpha-fetoprotein (AFP), tumor characteristics (e.g., number of tumors, tumor type, maximal tumor diameter, portal vein invasion, and bile duct invasion), mUICC stage, BCLC stage, and initial treatment modality. In this study, we additionally determined the location of extrahepatic spread by reviewing the imaging findings and electronic medical records, as the HCC registry lacked the detailed information. Tumor type was classified as either nodular or diffuse. The nodular tumor type comprised single to multiple discrete intrahepatic tumors whereas the diffuse tumor type represented huge tumor (at least 7 cm in diameter) with ill-defined edges [19]. Overall survival was defined as the time from diagnosis to the last follow-up or death, whichever occurred first. The referral date was May 31, 2018.

### Statistical analysis

All statistical analyses were conducted using SPSS 24 software package (IBM Corporation, Armonk, NC, USA). Data are expressed as the median (quartile), median (range), or number (%) of patients. The chi-square test, Fisher’s exact test, and Mann-Whitney test were used to compare the baseline characteristics and variables between the two groups. Survival analyses were performed using the Kaplan-Meier method with the differences in survival curves assessed using the log-rank test. Cox proportional hazards models were used to estimate the hazard ratios (HRs) for the variables. Any significant risk factors associated with overall survival were used in the stratified analysis comparing TACE and sorafenib. All analyses involved two-sided tests of significance with a P value less than 0.05 considered as statistically significant.

## Results

Table 1 shows the baseline characteristics of the study population and comparison based on the initial treatment modality. One hundred seventeen patients (62.9%) received TACE, and 69 patients (37.1%) underwent the initial treatment with sorafenib. Patients treated with sorafenib were younger, with higher AFP levels, higher proportion of diffuse/infiltrative tumor, more advanced portal vein invasion, and distant metastasis when compared with those that received TACE (Table 1).

**Table 1:** Baseline characteristics and comparison based on initial treatment modality.

Variables	Overall (N = 186)	TACE (N = 117)	Sorafenib (N = 69)	p value
Age	55 (49–63)	57 (51–65)	54 (48–59)	0.014
Male	160 (86.0)	100 (85.5)	60 (87.0)	0.83
Etiology				0.33
HBV*	152 (81.7)	93 (79.5)	59 (85.5)	
Others^†^	34 (18.3)	24 (20.5)	10 (14.5)	
Performance status (ECOG)				0.15
0	165 (88.7)	107 (91.5)	58 (84.1)	
≥1	21 (11.3)	10 (8.5)	11 (15.9)	
Child-Pugh class				0.52
A	155 (83.3)	100 (85.5)	55 (79.7)	
B	28 (15.1)	15 (12.8)	13 (18.8)	
C	3 (1.6)	2 (1.7)	1 (1.4)	
ALBI grade				0.33
1	72 (38.7)	50 (42.7)	22 (31.9)	
2	195 (56.5)	62 (53.0)	43 (62.3)	
3	9 (4.8)	5 (4.3)	4 (5.8)	
AFP, ng/ml	569 (33–13883)	252 (18–3837)	5655 (88–48595)	<0.001
<20	42 (22.6)	31 (26.5)	11 (15.9)	0.11
≥20	144 (77.4)	86 (73.5)	58 (84.1)	
Tumor type				0.015
Nodular	156 (83.9)	104 (88.9)	52 (75.4)	
Diffuse / infiltrative	30 (16.1)	13 (11.1)	17 (24.6)	
Maximal tumor diameter				0.94
<5 cm	41 (22.0)	26 (22.2)	15 (21.7)	
≥5 cm	145 (78.0)	91 (77.8)	54 (78.3)	
Portal vein invasion				0.042
No	69 (37.1)	47 (40.2)	22 (31.9)	
Segmental/lobar	83 (44.6)	55 (47.0)	28 (40.6)	
Main/bilateral	34 (18.3)	15 (12.8)	19 (27.5)	
Bile duct invasion	17 (9.1)	9 (7.7)	8 (11.6)	0.43
LN metastasis				0.015
No	48 (25.8)	23 (19.7)	25 (36.2)	
Yes	138 (74.2)	94 (80.3)	44 (63.8)	
Distant metastasis				<0.001
Lung	51 (27.3)	16 (13.6)	35 (50.7)	
Bone	14 (7.5)	7 (5.9)	7 (10.1)	
Adrenal gland	7 (3.7)	2 (1.7)	5 (7.2)	
Others^‡^	5 (2.7)	5 (4.2)	0 (0)	
Type of EHM				<0.001
LN	113 (60.8)	90 (76.9)	23 (33.3)	
Distant + LN metastases	25 (13.4)	4 (3.4)	21 (30.4)	
Distant metastasis only	48 (25.8)	23 (19.7)	25 (36.2)	

Data are presented as number (%) or median (quartile)

*One case in systemic therapy group had both HBV and HCV.

^†^Others include alcohol and non-B non-C.

^‡^Others include peritoneum, kidney, brain and rectovesical seeding.

Abbreviation: TACE, transarterial chemoembolization; Eastern Cooperative Oncology Group, ECOG; ALBI, albumin-bilirubin; AFP, alpha-fetoprotein; EHM, extrahepatic metastasis; LN, lymph node

Initial and subsequent treatment are summarized in Table 2. Of the 117 patients who underwent TACE, median number of TACE sessions were two. Twelve patients showed hepatic decompensation, defined by new-onset hyperbilirubinemia (>3 mg/dl) which was attributable to TACE. Three of them had significant hepatic dysfunction preventing further treatment, while the other nine patients recovered within an average of one week (range 3 to 21 days) of supportive care. Of the 69 patients who started sorafenib, median duration of sorafenib treatment was 1.5 months (range: 0.1–75.8 months), and 21 patients (30.4%) had dose reduction/interruption or permanent discontinuation related to sorafenib side effects. After initial treatment, sorafenib was used as a subsequent treatment in 26 patients (22.2%) among those who started treatment with TACE, while two patients (2.9%) received TACE among those who started treatment with sorafenib (Table 2).

**Table 2:** Initial and subsequent treatment according to initial treatment modality.

	**TACE (n = 117)**	**Sorafenib (n = 69)**
Number of total TACE sessions	2 (1–15)	N/A
One time	38 (32.5%)	N/A
Two times	23 (19.6%)	N/A
Three or more times	56 (47.9%)	N/A
Interval between TACE sessions (months)	1.4 (0.4–10)	N/A
Hepatic decompensation after TACE^†^	12 (10.2%)	
Recovered after supportive care	9 (7.7%)	
Not recovered after TACE	3 (2.6%)	
Median duration of sorafenib use (months)		1.5 (0.1–75.8)
Dose reduction/interruption		14 (20.3%)
Permanent discontinuation		7 (10.1%)
Other subsequent treatments		
Sorafenib	26 (22.2%)	N/A
TACE	N/A	2 (2.9%)
Radiotherapy	47 (40.2%)	4 (5.8%)
Others*	3 (2.6%)	4 (5.8%)

Data is expressed as median (range) or number (%). Abbreviation. TACE, transarterial chemoembolization; N/A, not applicable.

*Other treatment includes radiofrequency ablation, combination treatment with radiofrequency ablation and TACE, transarterial radioembolization, and clinical trials.

^†^Defined by new-onset hyperbilirubinemia (>3 mg/dl) after TACE session.

**Fig 1:**
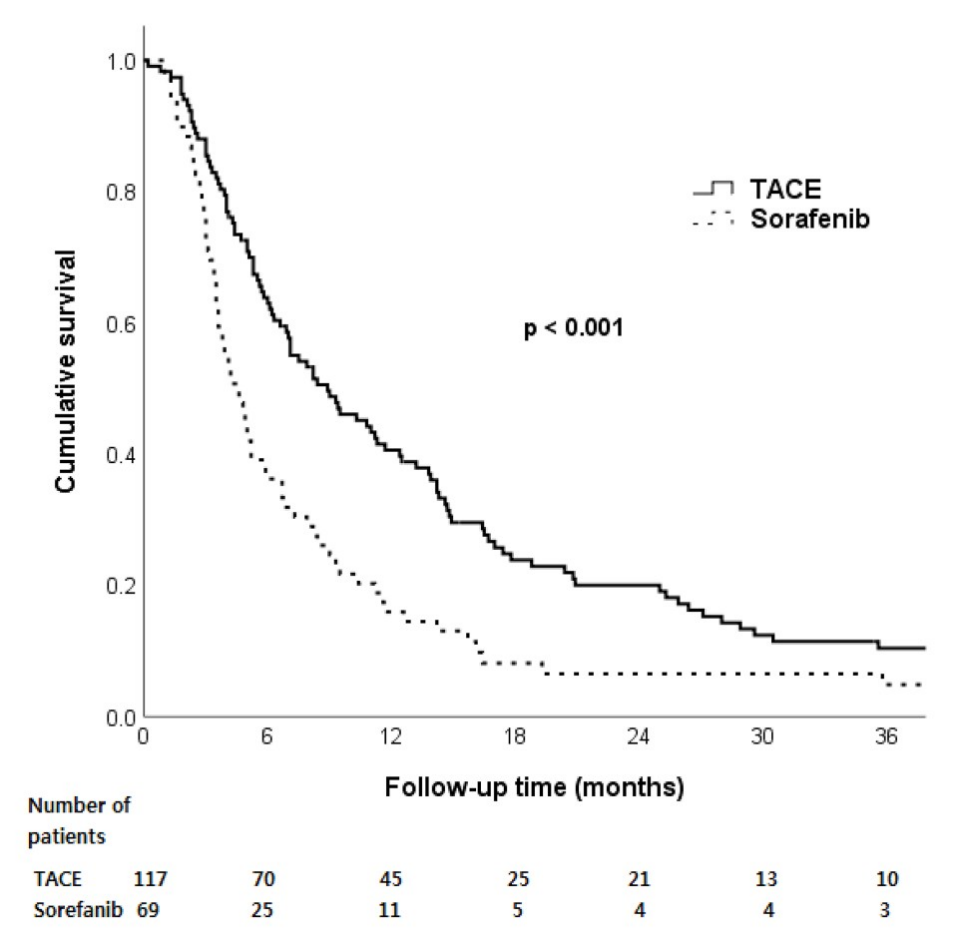
Overall survival according to initial treatment modality in hepatocellular carcinoma patients with extrahepatic spread. Abbreviation: TACE, transarterial chemoembolization.

During a median follow-up of 6.7 months (range: 0.2–94.6 months), mortality was observed in 168 patients (90.3%). Age, etiology, ALBI grade, AFP level, tumor type, portal vein invasion, type of EHM, and initial treatment modality were factors associated with overall survival in univariate analysis (Table 3). Median survival was higher in patients treated with TACE rather than sorafenib (8.2 months vs. 4.6 months, p < 0.001, Fig 1). However, in multivariable adjusted analysis, treatment modality was no longer an independent risk factor for overall survival (hazard ratio (HR): 1.19, 95% confidence interval (CI): 0.81–1.75, p = 0.36) (Table 3). The ALBI grade, portal vein invasion, and type of extrahepatic spread were independent factors associated with overall survival (Table 3).

**Table 3:** Risk factors for overall survival.

Variables	Un-adjusted		Multivariable	
	Hazard ratio (95% CI)	p value	Hazard ratio (95% CI)	p value
Age ≥ 60 (vs. <60), years	0.67 (0.48–0.93)	0.017	0.77 (0.54–1.11)	0.16
Female (vs. male)	0.86 (0.55–1.35)	0.51		
HBV (vs. others)	0.61 (0.41–0.91)	0.016	1.33 (0.86–2.07)	0.20
ECOG Performance status ≥1 (vs. 0)	1.00 (0.61–1.63)	0.99		
ALBI grade				
1	Reference		Reference	
2 to 3	1.62 (1.18–2.22)	0.003	1.54 (1.10–2.15)	0.011
AFP ≥20 ng/ml (vs. <20)	1.53 (1.06–2.21)	0.022	1.36 (0.92–2.00)	0.12
Diffuse tumor (vs. nodular)	2.26 (1.48–3.43)	<0.001	1.55 (0.95–2.53)	0.080
Tumor diameter ≥5 cm (vs. <5 cm)	1.34 (0.92–1.95)	0.13	0.98 (0.66–1.47)	0.94
Portal vein invasion				
No	Reference		Reference	
Segmental/Lobar	1.66 (1.17–2.34)	0.004	1.69 (1.19–2.41)	0.004
Main/bilateral	2.37 (1.53–3.67)	<0.001	2.18 (1.34–3.55)	0.002
Type of EHM				
LN	Reference		Reference	
Distant metastasis ± LN	1.84 (1.35–2.52)	<0.001	1.92 (1.34–2.76)	<0.001
Initial treatment modality				
TACE	Reference		Reference	
Sorafenib	1.77 (1.29–2.42)	<0.001	1.19 (0.81–1.75)	0.36

Abbreviation: TACE, transarterial chemoembolization; Eastern Cooperative Oncology Group, ECOG; ALBI, albumin-bilirubin; AFP, alpha-fetoprotein; EHM, extrahepatic metastasis: LN, lymph node

In subgroup analysis, TACE was associated with better overall survival than sorafenib in patients at a younger age, with HBV infection, higher AFP level, nodular tumor type, ALBI grade 2 to 3, segmental/lobar portal vein invasion, or nodal metastasis in un-adjusted analysis (Table 4). However, in multivariable adjusted model, TACE was associated with better survival than sorafenib, only for patients aged below 60 years, and patients with segmental/lobar portal vein invasion.

**Table 4:** Overall survival by initial treatment modality (transarterial chemoembolization vs. sorafenib) according to pre-defined subgroups.

Variables	Un-adjusted		Multivariable-adjusted	
	Hazard ratio (95% CI)	p value	Hazard ratio (95% CI)	p value
Age				
<60 (n = 126)	0.46 (0.32–0.68)	<0.001	0.58 (0.37–0.91)	0.017
≥60 (n = 60)	0.95 (0.51–1.77)	0.88	1.99 (0.91–4.38)	0.086
Etiology				
HBV (n = 152)	0.61 (0.43–0.85)	0.004	0.74 (0.49–1.12)	0.15
Others (n = 34)	0.51 (0.23–1.14)	0.10	2.27 (0.73–7.12)	0.16
AFP				
<20 ng/ml (n = 42)	0.78 (0.37–1.64)	0.52	1.52 (0.54–4.25)	0.43
≥20 ng/ml (n = 144)	0.54 (0.38–0.76)	<0.001	0.75 (0.49–1.16)	0.20
Tumor type				
Nodular (n = 156)	0.58 (0.41–0.82)	0.002	0.78 (0.53–1.17)	0.23
Diffuse (n = 30)	0.99 (0.46–2.13)	0.98	2.93 (0.69–12.43)	0.15
ALBI grade				
1 (n = 72)	0.61 (0.36–1.05)	0.076	0.92 (0.47–1.75)	0.79
2 to 3 (n = 114)	0.55 (0.37–0.82)	0.003	0.81 (0.48–1.33)	0.40
Portal vein invasion				
No (n = 69)	0.67 (0.39–1.15)	0.15	1.51 (0.78–2.91)	0.21
Segmental/lobar (n = 83)	0.49 (0.30–0.80)	0.004	0.44 (0.25–0.79)	0.006
Main/bilateral (n = 34)	0.50 (0.24–1.05)	0.067	0.76 (0.27–2.15)	0.61
Type of extrahepatic spread				
LN (n = 113)	0.58 (0.36–0.94)	0.028	0.70 (0.41–1.19)	0.19
Distant metastasis ± LN (n = 73)	0.82 (0.51–1.34)	0.44	1.01 (0.57–1.77)	0.98

Abbreviation: AFP, alpha-fetoprotein; ALBI, albumin-bilirubin; LN, lymph node

## Discussion

In this study, we analyzed a total of 186 consecutive HCC patients with EHM who were treated initially with TACE (n = 117) or sorafenib (n = 69) at a single tertiary hospital. Patients treated with TACE showed better overall survival compared with those receiving sorafenib. However, baseline characteristics varied significantly, and when adjusted, no significant difference existed in overall survival between TACE- or sorafenib-treated patients. In subgroup analysis, TACE was associated with better overall survival than sorafenib among younger patients (age < 60 years) and in patients with segmental/lobar portal vein invasion. Among the other subgroups, TACE was not a factor linked to improved overall survival.

To the best of our knowledge, no randomized controlled trials have investigated the efficacy and safety of TACE compared with those of sorafenib in HCC patients with EHM. Several observational studies are available, but with limitations. In a study involving 251 newly diagnosed HCC patients with EHM, repeated TACE showed survival benefit [14]; however, only a minor proportion received sorafenib treatment (n = 13). Another study involving 240 patients with HCC found intrahepatic tumor status as a significant predictor of survival [20]; however, it was composed of a heterogeneous population (141 patients with EHM at diagnosis and 99 patients who developed EHM during follow-up) who underwent a variety of treatment. Another study from Germany analyzed 215 patients with metastatic HCC and reported that treatment with intrahepatic TACE (n = 42) and a combination of TACE and sorafenib (n = 23) were associated with improved survival [15]. However, the reference group comprised those without therapy (n = 102), and not those that received sorafenib (n = 48) as initial treatment. In a study conducted at three German referral centers, TACE (n = 74) was not associated with better survival than sorafenib (n = 98) among HCC patients with EHM [21]. Several studies compared TACE and sorafenib in advanced-stage HCC [22–26] and reported different results. In these studies, the advanced stage was usually defined by BCLC stage C, including patients with or without EHM. In a study involving 382 advanced-stage HCC treated with TACE, those with EHM showed significantly worse survival compared with those without EHM, suggesting the presence of EHM means aggressive tumor biology [22]. Hence, studies comparing TACE and sorafenib in advanced-stage HCC should be carefully interpreted based on the presence of EHM.

In our study, survival was better in the TACE group; however the benefit disappeared after multivariable adjustment for age, etiology, ALBI grade, AFP level, tumor type, portal vein invasion, and type of EHM. This finding indicates that the better outcome observed in patients who received TACE was due to the selection of better patients upfront. Since it is widely accepted that liver function and tumor status have a strong influence on the prognosis of patients with HCC, it is important to preserve liver function [27–29]. In our study, the ALBI grade 2 or higher was associated with a significantly lower survival rate than the ALBI grade 1 (HR 1.54, 95% CI: 1.10–2.15, p = 0.011). Repeated exposure to TACE increases the risk of liver dysfunction [30]. In our series, 10.2% of patients experienced hepatic decompensation after TACE, and of those, three patients (2.6%) did not recover after TACE. Therefore, repeated TACE for HCC patients with EHM without strong evidence of the appropriate target group can lead to worse patient survival in this era of development of promising new systemic drugs. To determine whether the specific subgroups exist who may benefit from TACE, we performed a subgroup analysis. The analysis revealed that younger age (< 60 years) and portal vein invasion limited to segmental/lobar invasion showed favorable outcome following TACE compared with sorafenib. However, careful interpretation is needed as the definitive factors underlying the enhanced outcome in this subgroup are not clear. In a recent randomized controlled trial for advanced-stage HCC defined by macroscopic vascular invasion, TACE combined with radiotherapy showed better outcome than sorafenib [31], indicating that HCC with portal vein invasion may be ameliorated with TACE compared with sorafenib; however, in this trial, patients with EHM was excluded from the beginning [31]. In this study, the median duration of sorafenib use was 1.5 months. Sorafenib dose reduction, interruption or permanent discontinuation was observed in 30.4% of patients. Among patients who started treatment with TACE, 22.2% received subsequent treatment with sorafenib, while subsequent TACE treatment was observed in only 2 patients who started treatment with sorafenib. The subsequent treatment with sorafenib may have also had favorable effects on patient outcome among patients who started treatment with TACE. Taken together, performing TACE for intrahepatic tumor control in EHM does not appear to be an effective strategy in the era of sorafenib. Although it seems that TACE might be superior in certain patient subgroups, such as those younger and with segmental or lobar portal vein invasion, additional studies are warranted. In the future, with better systemic management options, the concept of intrahepatic tumor control in HCC patients with extrahepatic metastasis may require re-evaluation.

There are some limitations of this study. This is an observational study of patients treated according to physician’s direction, with possible unidentifiable bias favoring or excluding each treatment. In our series, majority of patients (76.9%) who underwent TACE had LN only metastasis, while LN only metastasis was observed in only 33.3% of patients who started sorafenib. The extent of intrahepatic or extrahepatic tumor control and the changes in liver function during treatment were not evaluated. This study was confined to a single referral center in a HBV-endemic area, limiting generalizability given the diverse etiology of HCC.

In summary, although the overall survival was better in HCC patients with EHM who received TACE compared with sorafenib, TACE was not associated with improved outcome in the multivariable adjusted model, suggesting that better outcomes were attributed to selection bias based on differences in baseline characteristics, and not from TACE. Although there can be specific subgroups who may benefit from TACE over sorafenib such as those younger and with segmental or lobar portal vein invasion, prospective well-designed studies are needed to confirm this finding. In the future, with the development and availability of more promising systemic agents, the concept of intrahepatic tumor control using TACE in HCC patients with EHM may no longer be necessary.

## Abbreviations

HCC: hepatocellular carcinoma
EHM: extrahepatic metastasis
TACE: transarterial chemoembolization
HR: hazard ratio
CI: confidence interval
ECOG: Eastern Cooperative Oncology Group
ALBI: albumin-bilirubin
AFP: alpha-fetoprotein
mUICC: modified Union for International Cancer Control
BCLC: Barcelona Clinic Liver Cancer
HBV: hepatitis B virus
